# A Sanatorium Protest

**Published:** 1920-06-26

**Authors:** 


### A Sanatorium Protest.

THE REAL ISSUE AT DANBURY.

THE decision of the Essex County Council to purchase a site at Danbury for a sanatorium has met with the familiar and hasty protest of local authorities, and the Danbury Parish Council has asked the Chelmsford Town Council also to resist the scheme. The Health Committee of the Town Council eventually recommended the Town Council to ask the Ministry of Health to disapprove of the sanatorium at Danbury because of the following reasons :—

The cost to the ratepayers of erecting a large institution at the present time. Alternatively it suggested that the Essex County Council should acquire the large building known as "Hylands," to be sold shortly, for conversion into a sanatorium at relatively cheap cost.

The Health Committee also condemned the erection of a sanatorium at Danbury because of the "adverse" effect on the village, one of the prettiest in Essex, and is largely frequented by visitors from Chelmsford.

We believe that on all grounds except economy the protest will prove here, as elsewhere, ill-founded. The conversion of an existing building, under the advice of a trained medical administrator, is better worth consideration.

